# Expression of telomerase-associated protein 1 and telomerase reverse transcriptase in hepatocellular carcinoma

**DOI:** 10.1054/bjoc.1999.1008

**Published:** 2000-02

**Authors:** N Toshikuni, K Nouso, T Higashi, H Nakatsukasa, T Onishi, T Kaneyoshi, Y Kobayashi, K Kariyama, K Yamamoto, T Tsuji

**Affiliations:** First Department of Internal Medicine, Okayama University Medical School, 2-5-1 Shikata-cho, Okayama-city, Okayama, 700-8558, Japan

**Keywords:** hepatocellular carcinoma, telomerase, hTERT, hTEP1

## Abstract

To know whether two protein components of human telomerase (human telomerase-associated protein 1 (hTEP1) and human telomerase reverse transcriptase (hTERT) are useful markers for telomerase activation in human liver diseases, we examined mRNA levels of these and telomerase activity in human liver samples. Twenty-three human hepatocellular carcinomas (HCCs) and corresponding adjacent livers were analysed for hTEP1 and hTERT expression by semiquantitative reverse transcription-polymerase chain reaction, and for telomerase activity by a telomeric repeat amplification protocol assay. Thirteen liver samples (ten HCCs and three dysplastic nodules) that were biopsied with 21-gauge needles were analysed for hTERT expression. hTEP1 was expressed in all samples examined. No correlation between hTEP1 expression and telomerase activity was observed. hTERT expression significantly correlated with telomerase activity (*P*< 0.001). The positivity of hTERT for HCC and corresponding non-cancerous liver was 100% and 30.4% respectively (*P*< 0.001). Seventy-four per cent (17/23) of HCCs showed strong hTERT expression, but none of the non-cancerous liver tissues did. hTERT expression of the 21-gauge needle biopsied specimens showed no significant difference from that of the surgical samples. The present study revealed that hTERT is strongly expressed in most HCCs, and that hTERT but not hTEP1 is a key component regulating telomerase activity in human liver. © 2000 Cancer Research Campaign


*British Journal of Cancer* (2000) **82**(4), 833–837
© 2000 Cancer Research Campaign
DOI: 10.1054/ bjoc.1999.1008, available online at http://www.idealibrary.com on IDEAL®

### Expression of telomerase-associated protein 1 and telomerase reverse transcriptase in hepatocellular carcinoma

**N Toshikuni, K Nouso, T Higashi, H Nakatsukasa, T Onishi, T Kaneyoshi, Y Kobayashi, K Kariyama, K Yamamoto and T Tsuji**

First Department of Internal Medicine, Okayama University Medical School, 2-5-1 Shikata-cho, Okayama-city, Okayama 700-8558, Japan.

**Summary** To know whether two protein components of human telomerase (human telomerase-associated protein 1 (hTEP1) and human telomerase reverse transcriptase (hTERT) are useful markers for telomerase activation in human liver diseases, we examined mRNA levels of these and telomerase activity in human liver samples. Twenty-three human hepatocellular carcinomas (HCCs) and corresponding adjacent livers were analysed for hTEP1 and hTERT expression by semiquantitative reverse transcription-polymerase chain reaction, and for telomerase activity by a telomeric repeat amplification protocol assay. Thirteen liver samples (ten HCCs and three dysplastic nodules) that were biopsied with 21-gauge needles were analysed for hTERT expression. hTEP1 was expressed in all samples examined. No correlation between hTEP1 expression and telomerase activity was observed. hTERT expression significantly correlated with telomerase activity ($P < 0.001$). The positivity of hTERT for HCC and corresponding non-cancerous liver was 100% and 30.4% respectively ($P < 0.001$). Seventy-four per cent (17/23) of HCCs showed strong hTERT expression, but none of the non-cancerous liver tissues did. hTERT expression of the 21-gauge needle biopsied specimens showed no significant difference from that of the surgical samples. The present study revealed that hTERT is strongly expressed in most HCCs, and that hTERT but not hTEP1 is a key component regulating telomerase activity in human liver. © 2000 Cancer Research Campaign

**Keywords:** hepatocellular carcinoma; telomerase; hTERT; hTEP1

Telomerase is a ribonucleoprotein that synthesizes the repeating sequence (TTAGGG) *n* in human chromosomal ends (Morin, 1989). This protein is thought to be essential for the acquisition of cellular immortality, because of its ability to overcome the reduction of chromosomal ends that occurs normally in somatic cells during cell divisions (Allsopp et al, 1992; Counter et al, 1992; Bodnar et al, 1998). After the advent of the telomeric repeat amplification protocol (TRAP) assay, telomerase activity was revealed in many human cancers, and its association with carcinogenesis as well as cellular immortality was postulated (Kim et al, 1994).

Human hepatocellular carcinoma (HCC) develops mainly in liver cirrhosis, which consists of numerous regenerative nodules. The existence of large regenerative nodules frequently makes the accurate diagnosis of small HCC difficult. Therefore, useful markers for the diagnosis of HCC have been sought. We have previously reported that telomerase activity was found in 84% of the HCCs examined, demonstrating the usefulness of examining telomerase activity in the differential diagnosis of HCC (Nouso et al, 1996).

Recently, three components of the telomerase were cloned; human telomerase RNA component (hTERC), human telomerase-associated protein 1 (hTEP1) and human telomerase reverse transcriptase (hTERT) (Feng et al, 1995; Harrington et al, 1997; Nakamura et al, 1997). Of the three components, hTERC expression has been observed in both telomerase-positive and -negative tissues, and no close correlation between the hTERC expression and the telomerase activity was reported, although hTERC contains the essential template region specifying the addition of telomerase sequence (Feng et al, 1995; Avilion et al, 1996). The other protein components are thought to be crucial for the regulation of telomerase activity, since hTERT has been proved to be the catalytic core protein component of telomerase and hTEP1 is a putative regulator domain of which post-translational modification is closely related with telomerase activity in the mouse homologue (Nakamura et al, 1997; Nakayama et al, 1997, 1998; Bodnar et al, 1998).

In the present study, we semiquantified the mRNA expressions of hTEP1 and hTERT, and the telomerase activity in human liver tissues to examine the regulatory mechanisms of telomerase in human liver diseases, and analysed whether these components are useful markers for telomerase activation in clinical samples including 21-gauge (21-G)-needle biopsied specimens.

### MATERIALS AND METHODS

### Patients and samples

Twenty-three surgically resected HCCs (11 well-, 11 moderately-, one poorly differentiated), and the corresponding adjacent non-cancerous liver tissues (ten liver cirrhosis, 13 chronic hepatitis) were analysed (Table 1). Of the 23 patients, three (13%) were female, and the patients' ages ranged from 38 to 75 years (mean = 62.7 years). Three patients (13%) were positive for hepatitis B virus surface antigen, 19 patients (82.6%) were positive for

*Received 19 July 1999*
*Revised 13 September 1999*
*Accepted 16 September 1999*

*Correspondence to:* K Nouso

**Table 1** Telomerase activity and expression of hTERT and hTEP1 in surgically resected HCC

| No.of cases | Age (years)/Sex | HCC Differentiation | HCC Size (cm) | Non-cancerous liver | AFP (ng ml⁻¹) | PIVKA-II (mAU ml⁻¹) | Telomerase T | Telomerase NT | hTERT T | hTERT NT | hTEP1[a] T | hTEP1[a] NT |
|---|---|---|---|---|---|---|---|---|---|---|---|---|
| 1 | 56/M | Moderate | 4.3 | LC (C) | 587 | 150 | ++ | + | ++ | + | 0.50 | 1.94 |
| 2 | 61/M | Moderate | 2.0 | CH (C) | 309 | 118 | ++ | – | ++ | – | 1.10 | 1.30 |
| 3 | 75/F | Moderate | 3.5 | CH (C) | 104 | 8 | ++ | – | ++ | – | 1.11 | 1.47 |
| 4 | 64/M | Well | 3.5 | CH (C) | 9.7 | 112 | + | + | ++ | + | 0.74 | 1.41 |
| 5 | 62/F | Well | 2.7 | LC (C) | 7.5 | 0 | ++ | – | ++ | + | 2.51 | 2.01 |
| 6 | 57/M | Well | 2.7 | CH (C) | 10.8 | 38 | + | – | + | – | 1.84 | 1.59 |
| 7 | 68/M | Well | 3.0 | LC (C) | 3.1 | 225 | + | – | + | – | 0.89 | 0.72 |
| 8 | 55/M | Poor | 10.0 | CH (B) | 40000 | 656 | + | – | ++ | – | 1.80 | 1.42 |
| 9 | 60/M | Well | 2.5 | LC (C) | 893 | 298 | ++ | – | ++ | – | 1.99 | 1.25 |
| 10 | 65/M | Moderate | 4.5 | LC (C) | 443 | 0 | ++ | – | ++ | – | 1.54 | 1.26 |
| 11 | 42/M | Well | 5.0 | LC (B) | 790 | 15 | ++ | – | ++ | + | 1.60 | 1.46 |
| 12 | 38/M | Moderate | 2.5 | CH (B) | 11487 | 1914 | – | – | + | – | 1.31 | 0.95 |
| 13 | 71/M | Moderate | 3.0 | CH (C) | 2111 | 86 | ++ | – | ++ | + | 1.07 | 1.60 |
| 14 | 72/M | Well | 1.5 | CH (C) | 26.3 | NI | ++ | + | + | – | 1.31 | 1.38 |
| 15 | 65/M | Well | 2.0 | CH (C) | 6.1 | 16 | ++ | + | ++ | – | 1.38 | 2.18 |
| 16 | 67/M | Moderate | 2.2 | LC (C) | 11.4 | 15 | ++ | – | ++ | – | 1.10 | 1.32 |
| 17 | 68/M | Moderate | 2.0 | LC (NBNC) | 14 | 60 | ++ | – | ++ | – | 1.43 | 1.31 |
| 18 | 66/M | Well | 1.8 | CH (C) | 4.5 | 0 | ++ | – | ++ | – | 1.38 | 1.42 |
| 19 | 70/M | Moderate | 1.8 | CH (C) | 2.5 | <63 | ++ | + | ++ | + | 0.54 | 1.49 |
| 20 | 68/M | Moderate | 1.6 | CH (C) | 211 | <70 | + | – | ++ | – | 0.84 | 0.93 |
| 21 | 64/M | Well | 2.0 | CH (C) | 20.8 | 0 | + | + | + | + | 1.17 | 1.08 |
| 22 | 62/F | Well | 3.0 | LC (C) | 18.4 | 0 | + | – | + | – | 1.18 | 1.13 |
| 23 | 67/M | Moderate | 4.5 | LC (C) | 29.8 | NI | ++ | – | ++ | – | 1.00 | 0.98 |

HCC: hepatocellular carcinoma; AFP: alpha-fetoprotein; PIVKA-II: protein induced by vitamin K absence-II; hTERT: human telomerase reverse transcriptase; hTEP1: human telomerase-associated protein 1; T: tumorous portion; NT: non-tumorous portion; M: male; F: female; CH: chronic hepatitis; LC: liver cirrhosis; B: positive for hepatitis B virus surface antigen; C: positive for hepatitis C virus antibody; NBNC: non-B, non-C; NI: not informative; –: negative; +: weak; ++: strong. [a]hTEP1 mRNA was expressed as the ratio to 18S rRNA product.

**Table 2** hTERT expression in liver tumours with biopsied with 21-gauge fine needle

| No. of cases | Age (years)/Sex | No. of samples | Liver tumour Histology | Liver tumour Size (cm) | Non-tumorous liver | hTERT mRNA in tumour |
|---|---|---|---|---|---|---|
| 1 | 74/M | 1 | HCC (Moderate) | 1.3 | CH (C) | + |
| 2 | 70/M | 2 | HCC (Moderate) | 1.4 | LC (C) | ++ |
| 3 | 64/F | 3 | DN | 1.9 | LC (C) | + |
| 4[a] | 76/M | 4 | HCC (Moderate) | 2.3 | CH (C) | ++ |
| | | 5 | HCC (Well) | 1.7 | | ++ |
| 5 | 65/M | 6 | HCC (Well) | 1.1 | CH (C) | ++ |
| 6 | 65/F | 7 | HCC (Well) | 1.0 | CH (C) | ++ |
| 7 | 60/M | 8 | HCC (Well) | 3.1 | CH (C) | ++ |
| 8 | 67/M | 9 | HCC (Moderate) | 1.6 | LC (C) | + |
| 9 | 58/F | 10 | DN | 1.5 | CH (C) | ++ |
| 10 | 75/M | 11 | HCC (Well) | 1.0 | LC (NBNC) | + |
| 11 | 68/F | 12 | DN | 1.2 | LC (C) | + |
| 12 | 74/F | 13 | HCC (Moderate) | 4.0 | LC (C) | ++ |

hTERT: human telomerase reverse transcriptase; M: male; F: female; HCC: hepatocellular carcinoma; DN: dysplastic nodule; CH: chronic hepatitis; LC: liver cirrhosis; C: positive for hepatitis C virus antibody; NBNC: negative for hepatitis B virus surface antigen, negative for hepatitis C virus antibody; +: weak; ++: strong. [a]Case 4 had two liver tumours at the time of biopsy.

hepatitis C virus antibody, and the remaining patient was negative for both viral markers. Alpha-fetoprotein (AFP) and protein induced by vitamin K absence-II (PIVKA-II) were measured by enzyme immunoassay. The normal ranges of AFP and PIVKA-II are less than 10 ng $ml^{-1}$ and 100 mAU $ml^{-1}$ respectively. Each tissue sample was bisected; half of the tissue was examined for histological diagnosis, and the other half was stored at –80°C until used for reverse transcription-polymerase chain reaction (RT-PCR) and TRAP assay. Thirteen tumour samples (ten HCCs and three dysplastic nodules) were obtained by aimed tumour biopsies with 21-G needles (Table 2). The tumours were biopsied twice and analysed for both histological diagnosis and hTERT mRNA expression. The histological diagnosis of liver tumours was made according to the criteria outlined by International Working Party (1995). Informed consent was obtained from all patients for the experimental use of the samples.

*© 2000 Cancer Research Campaign*

### cDNA preparation and semiquantitative RT-PCR

Total RNA was extracted with RNA zol™ (TEL-TEST, Friendswood, TX, USA) according to the manufacturer's protocol. cDNA was generated from 3 μg of RNA by reverse transcriptase (RAV2, Takara, Shiga, Japan) with random hexamers as primers. To ensure that the RNA was not degraded, a PCR assay for 30 cycles (94°C, 1 min; 60°C, 1 min; 72°C, 1 min) with primers specific for glyceraldehyde-3-phosphate dehydrogenase (GAPDH) was carried out on each cDNA sample.

hTEP1 expression was quantified with the QuantumRNA™ module (Ambion, Austin, TX, USA), which uses 18S rRNA as an internal control (Raeymaekers, 1995). AmpliTaq Gold™ (Perkin-Elmer Applied Biosystems Japan, Chiba, Japan) was used as a DNA polymerase to increase the fidelity of the PCR by a hot start. Briefly, cDNA was amplified with TCAAGCCAAACCTGAATCTGAG (residues 7483–7504) as a sense and CCCGAGTGAATCTTTC-TACGC (residues 7726–7746) as an antisense primer in the presence of 18S PCR Competimers™ (Ambion) and the 18S PCR primer pair at the ratio of 4:6 to optimize the amplification product of 18S rRNA (Nakamura et al, 1997). The PCR conditions were preheating at 95°C for 12 min, followed by 35 PCR cycles (95°C, 1 min; 54°C, 1 min; 72°C, 1 min), and a final extension at 72°C for 4 min. The amplification was in the exponential range (data not shown). The PCR products were electrophoresed in 1% agarose gels and stained with ethidium bromide. The intensity of the bands was quantified by a charge-coupled device image sensor (Analytical Imaging Station, Imaging Research, Ontario, Canada), and hTEP1 expression was expressed as the ratio to the 18S rRNA product.

We semiquantified hTERT by changing PCR cycle numbers because the difference of the expression levels among samples was too large to use the QuantumRNA™ module. hTERT was amplified by AmpliTaq Gold™ with a sense primer CGGAAGAGTGTCTG-GAGCAA (residues 1784–1803) and an antisense primer GGAT-GAAGCGGAGTCTGGA (residues 1910–1928) (Nakamura et al, 1997). The PCR conditions were preheating at 95°C for 12 min, followed by 38 or 45 PCR cycles (95°C, 1 min; 52°C, 1 min; 72°C, 1 min 30 s) and a final extension at 72°C for 4 min. We used low annealing temperature to increase the sensitivity. The PCR products were electrophoresed in 1% agarose gels and stained with ethidium bromide. We chose 38 PCR cycles for the detection of hTERT although the products could be detected at 32 cycles in some samples, because we could effectively differentiate the samples with strongly positive telomerase activity from those with weakly positive telomerase activity at 38 cycles in a preliminary experiment. The samples that were positive at 38 PCR cycles, positive at 45 PCR cycles, and negative at 45 PCR cycles were denoted as strong (++), weak (+), and negative (–) respectively.

We confirmed that no contamination of the genomic DNA existed by treating RNA samples with deoxyribonuclease before RT-PCR.

### Telomeric repeat amplification protocol assay

The telomerase activity in the tissues was analysed by a TRAP assay as previously described (Nouso et al, 1996). Extracted samples that were positive at 0.6 μg (24-h exposure), positive at 6 μg (48-h exposure), and negative at 6 μg (48-h exposure) were denoted as strong (++), weak (+), and negative (–) respectively. Rat myogenin sequence was used as an internal standard to detect the existence of an inhibitor of TRAP assay.

### Statistical analyses

All experiments were done in duplicate and the reproducibility was confirmed. Statistical analyses were performed with $\chi^2$ test, Student's *t*-test, and Fisher's exact probability test. A *P*-value of $P < 0.05$ was considered significant.

### RESULTS

The transcripts of hTEP1 and hTERT and the telomerase activity were examined (Figure 1 and Table 1). Telomerase activity was detected in 22 of 23 HCC samples and six of 23 non-cancerous liver tissues. Sixty-five per cent (15/23) of HCCs showed strong telomerase activity, but none of the non-cancerous tissues did. No evidence of an inhibitor in TRAP assay was proved in all samples examined.

hTEP1 mRNA was expressed in all samples. The expression was not significantly different among the chronic hepatitis, liver cirrhosis, and HCC samples (Table 3). The difference of tumour size and viral markers did not correlate with hTEP1 expression; however, the hTEP1 expression in the well-differentiated HCC was higher than that in the moderately differentiated HCC ($P < 0.05$, Student's *t*-test). The hTEP1 expression in the samples with negative, weak, and strong telomerase activity was

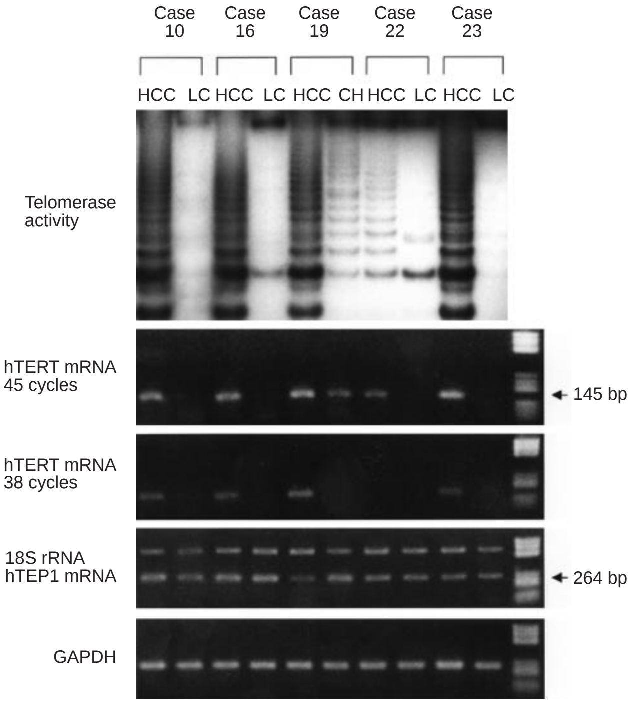

**Figure 1** The expression of telomerase activity, human telomerase-associated protein 1 (hTEP1), human telomerase reverse transcriptase (hTERT), and glyceraldehyde-3-phosphate dehydrogenase (GAPDH) in five patients with hepatocellular carcinoma (HCC). The telomerase activity was analysed by a telomeric repeat amplification assay. hTEP1 mRNA (lower band) was amplified by RT-PCR and compared with the band intensities of 18S rRNA (upper band). Two different PCR cycles (38 and 45) were used for the semiquantitation of hTERT mRNA. Note that the telomerase activity correlated with hTERT but not with hTEP1. CH: chronic hepatitis; LC: liver cirrhosis

© 2000 Cancer Research Campaign

**Table 3** hTEP1 expression in HCC and corresponding non-cancerous liver

| | *n* | hTEP1 mRNA/18S rRNA (mean ± standard deviation) |
|---|---|---|
| HCC | 23 | 1.28 ± 0.46 |
| Differentiation | | |
| Well | 11 | 1.44 ± 0.48[a] |
| Moderate | 11 | 1.05 ± 0.31[a] |
| Poor | 1 | 1.80 |
| Size (cm) | | |
| ≤2 | 8 | 1.14 ± 0.29 |
| 2≤3 | 8 | 1.49 ± 0.53 |
| >3 | 7 | 1.18 ± 0.45 |
| Viral marker | | |
| B | 3 | 1.57 ± 0.20 |
| C | 19 | 1.22 ± 0.48 |
| NBNC | 1 | 1.43 |
| Non-cancerous liver | 23 | 1.37 ± 0.34 |
| CH | 13 | 1.40 ± 0.31 |
| LC | 10 | 1.35 ± 0.38 |

hTEP1: human telomerase-associated protein 1; HCC: hepatocellular carcinoma; B: positive for hepatitis B virus surface antigen; C: positive for hepatitis C virus antibody; NBNC: non-B, non-C; CH: chronic hepatitis; LC: liver cirrhosis. [a]$P < 0.05$ (Student's *t*-test).

**Table 5** hTERT expression in HCC and corresponding non-cancerous liver

| | hTERT mRNA | | | |
|---|---|---|---|---|
| | – | + | ++ | Positive no. (%) |
| HCC | 0 | 6 | 17 | 23/23 (100)[a] |
| Differentiation | | | | |
| Well | 0 | 5 | 6 | |
| Moderate | 0 | 1 | 10 | |
| Poor | 0 | 0 | 1 | |
| Size (cm) | | | | |
| ≤2 | 0 | 2 | 6 | |
| 2≤3 | 0 | 4 | 4 | |
| >3 | 0 | 0 | 7 | |
| Viral marker | | | | |
| B | 0 | 1 | 2 | |
| C | 0 | 5 | 14 | |
| NBNC | 0 | 0 | 1 | |
| Non-cancerous liver | 16 | 7 | 0 | 7/23 (30.4)[a] |
| CH | 9 | 4 | 0 | 4/13 (30.8) |
| LC | 7 | 3 | 0 | 3/10 (30) |

hTERT: human telomerase reverse transcriptase; HCC: hepatocellular carcinoma; –: negative; +: weak; ++: strong; B: positive for hepatitis B virus surface antigen; C: positive for hepatitis C virus antibody; NBNC: non-B, non-C; CH: chronic hepatitis; LC: liver cirrhosis. [a]$P < 0.001$ ($\chi^2$ test).

**Table 4** Relationship between hTERT and telomerase activity in 23 patients with HCC

| | | Telomerase activity[a] | | |
|---|---|---|---|---|
| | | – | + | ++ |
| hTERT[a] mRNA | – | 14 | 2 | 0 |
| | + | 4 | 8 | 1 |
| | ++ | 0 | 3 | 14 |

hTERT: human telomerase reverse transcriptase; HCC: hepatocellular carcinoma; –: negative; +: weak; ++: strong. [a]$P < 0.001$ ($\chi^2$ test).

1.30 ± 0.29 ($n$ = 18), 1.38 ± 0.29 ($n$ = 13), and 1.30 ± 0.49 ($n$ = 15) respectively (mean ± standard deviation), and no significant difference was observed among the groups (Student's *t*-test).

In contrast to hTEP1, the expression of hTERT mRNA was closely related with the telomerase activity ($P < 0.001$, $\chi^2$ test, Table 4). The positivity of hTERT for the HCC and the corresponding non-cancerous liver was 100% and 30.4% respectively ($P < 0.001$, $\chi^2$ test, Table 5). Seventy-four per cent (17/23) of HCCs showed strong hTERT expression, whereas none of the non-cancerous tissues did. Although no significant difference was observed, the incidence of the strong expression in the moderately differentiated HCC tended to be higher than that in the well-differentiated HCC ($P = 0.074$, Fisher's test), and the incidence also tended to be higher in the HCC larger than 3 cm ($P = 0.079$, Fisher's test). Seventy-five per cent (6/8) of small HCCs (2 cm or less in diameter) showed strong hTERT expression. The viral marker status of HCC did not affect the positivity.

The sensitivity and the specificity of strong hTERT for the diagnosis of HCC was 73.9% (17/23) and 100% (23/23) respectively. In the 21 informative samples of which the following three markers were examined, the positivity of AFP, PIVKA-II and strong hTERT was 71.4% (15/21), 33.3% (7/21), and 76.2% (16/21) respectively (Table 1). The strong expression of hTERT was observed in 83.3% (5/6) of AFP-negative HCC and in 78.6% (11/14) of PIVKA-II-negative HCC.

The expression of hTERT was observed in all biopsied samples examined (Table 2). The incidence of strong hTERT expression in HCCs and in dysplastic nodules was 70% (7/10) and 33.3% (1/3) respectively.

### DISCUSSION

In the present study, we, for the first time, semiquantified hTEP1, hTERT, and telomerase activity simultaneously in human HCC with corresponding non-cancerous liver. We found that the expression of hTERT but not hTEP1 correlates significantly with the telomerase activity in the human liver. Our result is consistent with previous in vitro experiments and recent reports on human livers including a semiquantitation study of telomerase and hTERT with real-time PCR (Nakamura et al, 1997; Bodnar et al, 1998; Nakayama et al, 1998; Hisatomi et al, 1999).

The diagnosis of HCC has been difficult though imaging modalities have been drastically improved. Small liver nodules are now frequently found, but many are still difficult to obtain accurate diagnosis. For the diagnosis of small HCC, the most reliable method currently used is a histological examination of the biopsied samples, which is, however, sometimes ambiguous. In the present study, the high expression rate of strong hTERT in small HCC and the low incidence of strong hTERT expression in non-cancerous liver were observed. In addition, the high incidence of strong hTERT expression was observed even in serum AFP-negative or PIVKA-II-negative HCC; AFP and PIVKA-II are clinically used as good markers for the diagnosis of HCC (Nakagawa et al, 1999). These findings are all beneficial for the hTERT examination in the differential diagnosis of HCC. However, one of the three dysplastic nodules showed strong hTERT expression. Moreover, it has been reported in a recent study that dysplastic nodules in human livers exhibited telomerase activity at various levels (Hytiroglou et al, 1998). The distinction between HCCs and

 © 2000 Cancer Research Campaign dysplastic nodules is clinically important but still remains difficult. Since the number of dysplastic nodules examined was small, further investigation will be needed to clarify whether hTERT expression in dysplastic nodules differs from that in HCC.

In the evaluation of hTERT expression for diagnosing HCC, one has to be careful about the 'false-positive' of hTERT in non-cancerous liver. There are several possible reasons for the hTERT expression in non-cancerous liver. We have detected weak hTERT expression in peripheral blood mononuclear cells (data not shown). Infiltrating lymphocytes in chronic hepatitis or cirrhotic liver may be responsible for the hTERT expression in liver tissues. A harbouring micrometastasis of HCC may be another possibility, although the hTERT expression in surrounding non-cancerous liver was not related with the differential stage of the corresponding tumour in the present series.

hTEP1 has been shown to interact with mammalian telomerase RNA and telomerase activity (Harrington et al, 1997). However, no significant difference in the hTEP1 expression was observed between the telomerase-positive and -negative liver samples in the present study. Therefore, the measurement of hTEP1 expression is not applicable in the differential diagnosis of HCC. A modification of rat homologue of hTEP1 from p240 to p230 in accordance with the telomerase activation was reported, so that the examinations of the change of the molecular weight of hTEP1 might help the diagnosis of HCC (Nakayama et al, 1997).

We observed individual variations of hTEP1 expression and found that the moderately differentiated HCC expressed less hTEP1 than the well-differentiated HCC. Although this seems to be compatible with the report that hTEP1 expression in HL60 cells was augmented by the induction of differentiation, the reason for the difference in HCC is presently unknown (Reichman et al, 1997).

The detection of telomerase-active cells in the tissue section has been difficult, because most of the biopsied samples have been fixed with formalin and embedded in paraffin for the histological examination. Recently, a modified in situ hybridization method for detecting hTERT mRNA using formalin-fixed paraffin-embedded tissue was developed (Kolquist et al, 1998). Further histological analyses of hTERT may enable us to identify telomerase-active cells in human liver and provide us with new information about the accurate diagnosis of HCC.

### ACKNOWLEDGEMENTS

This work was supported by grants from the Intractable Liver Diseases Research Committee, the Japanese Ministry of Health and Welfare, and from the Japanese Ministry of Education (10670474). The authors thank First Department of Surgery, Okayama University Medical School and Dr Kazuhiko Morii (Himeji Red Cross Hospital) for providing a portion of the human liver samples.

### REFERENCES

Allsopp RC, Vaziri H, Patterson C, Goldstein S, Younglai EV, Futcher AB, Greider CW and Harley CB (1992) Telomere length predicts replicative capacity of human fibroblasts. *Proc Natl Acad Sci USA* **89:** 10114–10118

Avilion AA, Piatyszek MA, Gupta J, Shay JW, Bacchetti S and Greider CW (1996) Human telomerase RNA and telomerase activity in immortal cell lines and tumor tissues. *Cancer Res* **56:** 645–650

Bodnar AG, Ouellette M, Frolkis M, Holt SE, Chiu CP, Morin GB, Harley CB, Shay JW, Lichtsteiner S and Wright WE (1998) Extension of life-span by introduction of telomerase into normal human cells. *Science* **279:** 349–352

Counter CM, Avilion AA, LeFeuvre CE, Stewart NG, Greider CW, Harley CB and Bacchetti S (1992) Telomere shortening associated with chromosome instability is arrested in immortal cells which expresss telomerase activity. *EMBO J* **11:** 1921–1929

Feng J, Funk WD, Wang SS, Weinrich SL, Avilion AA, Chiu CP, Adams RR, Chang E, Allsopp RC, Yu J, Le S, West MD, Harley CB, Andrews WH, Greider CW and Villeponteau B (1995) The RNA component of human telomerase. *Science* **269:** 1236–1241

Harrington L, McPhail T, Mar V, Zhou W, Oulton R, Amgen EST program, Bass MB, Arruda I and Robinson MO (1997) A mammalian telomerase-associated protein. *Science* **275:** 973–977

Hisatomi H, Nagao K, Kanamaru T, Endo H, Tomimatsu M and Hikiji K (1999) Levels of telomerase catalytic subunit mRNA as a predictor of potential malignancy. *Int J Oncol* **14**: 727–732

Hytiroglou P, Kotoula V, Thung SN, Tsokos M, Fiel MI and Papadimitriou CS (1998) Telomerase activity in precancerous hepatic nodules. *Cancer* **82**: 1831–1838

International Working Party (1995) Terminology of nodular hepatocellular lesions. *Hepatology* **22**: 983–993

Kim NW, Piatyszek MA, Prowse KR, Harley CB, West MD, Ho PLC, Coviello GM, Wright WE, Weinrich SL and Shay JW (1994) Specific association of human telomerase activity with immortal cells and cancer. *Science* **266**: 2011–2015

Kolquist KA, Ellisen LW, Counter CM, Meyerson M, Tan LK, Weinberg RA, Haber DA and Gerald WL (1998) Expression of TERT in early premalignant lesions and a subset of cells in normal tissues. *Nat Genet* **19**: 182–186

Morin GB (1989) The human telomere terminal transferase enzyme is a ribonucleoprotein that synthesizes TTAGGG repeats. *Cell* **59**: 521–529

Nakagawa T, Seki T, Shiro T, Wakabayashi M, Imamura M, Itoh T, Tamai T, Nishimura A, Yamashiki N, Matsuzaki K, Sakaida N, Inoue K and Okamura A (1999) Clinicopathologic significance of protein induced vitamin K absence or antagonist II and α-fetoprotein in hepatocellular carcinoma. *Int J Oncol* **14**: 281–286

Nakamura TM, Morin GB, Chapman KB, Weinrich SL, Andrews WH, Linger J, Harley CB and Cech TR (1997) Telomerase catalytic subunit homologs from fission yeast and human. *Science* **277**: 955–959

Nakayama J, Saito M, Nakamura H, Matsuura A and Ishikawa F (1997) TLP1: a gene encoding a protein component of mammalian telomerase is a novel member of WD repeats family. *Cell* **88**: 875–884

Nakayama J, Tahara H, Tahara E, Saito M, Ito K, Nakamura H, Nakanishi T, Tahara E, Ide T and Ishikawa F (1998) Telomerase activation by hTRT in human normal fibroblasts and hepatocellular carcinomas. *Nat Genet* **18**: 65–68

Nouso K, Urabe Y, Higashi T, Nakatsukasa H, Hino N, Ashida K, Kinugasa N, Yoshida K, Uematsu S and Tsuji T (1996) Telomerase as a tool for the differential diagnosis of human hepatocellular carcinoma. *Cancer* **78**: 232–236

Raeymaekers L (1995) A commentary on the practical applications of competitive PCR. *Gen Res* **5**: 91–94

Reichman TW, Albanell J, Wang X, Moore MAS and Studzinski GP (1997) Downregulation of telomerase activity in HL60 cells by differentiating agents is accompanied by increased expression of telomerase-associated protein. *J Cell Biochem* **67**: 13–23

© 2000 Cancer Research Campaign
